# Supplementary material for: Mapping the self-generated magnetic fields due to thermal Weibel instability
Source: Proc Natl Acad Sci U S A. 2022 Dec 5;119(50):e2211713119. doi: 10.1073/pnas.2211713119 (PMC9897480; doi:10.1073/pnas.2211713119)
Supplement: Supplementary file 1 — Appendix 01 (PDF) [file pnas.2211713119.sapp.pdf]

PNAS

Supplementary Information for

Mapping the self-generated magnetic fields due to thermal Weibel instability

Chaojie Zhang[1, *], Yipeng Wu[1, †], Mitchell Sinclair[1], Audrey Farrell[1], Kenneth A. Marsh[1], Irina Petrushina[2], Navid Vafaei-Najafabadi[2,3], Apurva Gaikwad[2], Rotem Kupfer[3], Karl Kusche[3], Mikhail Fedurin[3], Igor Pogorelsky[3], Mikhail Polyanskiy[3], Chen-Kang Huang[4], Jianfei Hua[5], Wei Lu[5], Warren B. Mori[1,6], Chan Joshi[1, ‡]

* Chaojie Zhang, Yipeng Wu, Chan Joshi
Email: chaojiez@ucla.edu, wuyipeng@ucla.edu, cjoshi@ucla.edu

This PDF file includes:

Supplementary text
Figures S1 to S6
Legends for Movies S1 to S3

Other supplementary materials for this manuscript include the following:

Movies S1 to S3

Supplementary Information Text

Time jitter of the e- probe with respect to the CO_2 laser

In the experiment, the e- probe was synchronized to the CO_2 laser and the time jitter between the two was estimated by recording the propagation of the CO_2 ionization front in an underdense plasma using the e- probe. A dataset is shown in Fig. S1 where (a)-(d) show four shots where the delay of the CO_2 laser was changed using a translation stage. The white arrows mark the position of the ionization front. In Fig. S1e, the ionization front location is plotted as a function of the CO_2 delay. The orange line shows a linear fit to the data. From the fit we extracted the propagation velocity of the ionization front to be $v \approx 0.92 \pm 0.14c$. The group velocity of the CO_2 laser depends on the plasma density n_p, namely, $v_g = c\sqrt{1 - n_p/n_c}$ where $n_c \approx 1.3 \times 10^{19}$ cm^{-3} is the critical density for the CO_2 laser. Using the measured propagation velocity, we can estimate the plasma density to be $\sim 2 \times 10^{18}$ cm^{-3} which is in good agreement with the density $(1.8 \pm 0.2) \times 10^{18}$ cm^{-3} measured using the ionization induced plasma grating method (see later). The linear fit in Fig. S1e gives the position of CO_2 laser for each delay. The actual data point differs from this line because of the time jitter between the laser and the electron probe. For instance, if the electron probe comes later than it should in the (ideal) no-jitter case, the ionization front of the CO_2 laser has moved further to the left in the frame. Therefore, the difference between the blue dots (measured) and the orange line (estimated) ionization front in Fig. S1e, after being converted to time, gives the arrival time of the electron probe with respect to the CO_2 laser, which is plotted in Fig. S1f. Here a positive arrival time means the electron probe comes later than in the no-jitter case. Using this dataset, the RMS jitter between the CO_2 laser and the electron probe is calculated to be $\sigma \approx 0.4$ ps.

Maxwellian fitting residuals

The EVDs deduced from 3D PIC simulation are shown by the blue curves in Fig. 1C. These EVDs can be well fitted by Maxwellian distributions with $r^2 > 0.997$. The fitting residuals are shown in Fig. S2 to further illustrate the goodness of fit. In all three cases, for the major part of the distribution, the deviation of the EVD from a Maxwellian distribution is less than 0.05. In other words, the plasma indeed has the required temperature anisotropy for driving the Weibel instability.

Retrieve the k-dependent growth rates

Using the time-resolved measurements shown in Fig. 3C and D, we have retrieved the *k*-dependent growth rates of the B_x and B_y components. These are shown in Fig. 3E and F, respectively. In Figs. S3 and S4 we show the measured growth of the magnetic field components and the exponential fit

to the data for several representative wavevectors. In each subplot of Fig. S3, the blue circles represent the measured B_x field component (on log scale) with a specific wavevector k_y. The data shows that the magnetic field grows rapidly and then reaches saturation very quickly within a few ps. By assuming an exponential growth, we have fitted the data using the first two points to extract the growth rate. The fitting curve is shown by the red dashed line in each subplot. The retrieved *k*-dependent growth rate for B_x is shown by the blue curve in Fig. 3E. It's important to remember that the dynamic range of our data is less than a factor of 30. This means that if one is interested in collecting data about the saturated value of the magnetic field, the number of data points leading up to that saturated value are going to be limited to two or at most three. This is why in Fig.S3 and S4 below the growth rate has to be derived from mostly two values of magnitude of $B_{x,y}$ vs. time. A similar analysis was done for the B_y field and the results are plotted in Fig. S4. The retrieved k-dependent growth rate for B_x is shown by the orange curve in Fig. 3F.

As we have mentioned in the main text, it is also possible to extract the *k*-resolved growth rate for B_x by doing an intraframe analysis of the measured density directly. In the 3.3 ps frame (shown in Fig. 1D), the density strips are quasi-parallel to the horizontal direction, which implies that they are predominantly caused by the probe electrons being deflected by the B_x field. Here we assume that each column in the image of the measured modulated probe flux is caused by the 1D B_x field and neglect the contribution of B_y. At each horizontal position x, the local B_x field causes deflections of probe electrons with an angle distribution of $\theta_y \approx -\frac{e\int B_x dz}{\gamma m_e c}$. For the quasi-parallel probe beam used in the experiment, such an angle deflection will develop into a displacement of the electrons, $y = y_0 + \theta_y L$ after a propagation distance L, which corresponds to a modulated probe flux distribution

$$n = \frac{n_0}{|1 + (\partial\theta_y/\partial y_0)L|}$$

here n_0 is unperturbed probe flux distribution (background), y_0 is the original position of the undeflected electron. In the small-deflection regime where $\frac{\partial\theta_y}{\partial y_0}L > -1$, the measured probe flux distribution is uniquely determined by the deflection angle and the magnetic field,

$$\frac{1}{L}\left(\frac{n_0}{n} - 1\right) = \frac{\partial\theta_y}{\partial y_0} = -\frac{eW}{\gamma m_e c}\frac{\partial\bar{B}_x}{\partial y_0}$$

where we have used $\int B_x dz = \bar{B}_x W$, here W is the width of the field and $\bar{B}_x$ is the average field. Taking the Fourier analysis, we have

$$\mathrm{FFT}\left[\frac{1}{L}\left(\frac{n_0}{n} - 1\right)\right] = -\frac{eW}{\gamma m_e c}\mathrm{FFT}\left[\frac{\partial\bar{B}_x}{\partial y_0}\right] = -\frac{eW}{\gamma m_e c} i k_y \bar{B}_x(k_y)$$

This means that the k_y spectrum of $\bar{B}_x$ at each x position can be calculated by taking the Fourier analysis of $\frac{n_0}{n} - 1$, which is determined from measurements.

Figure S5 summarizes the analysis procedure. The measured probe flux distribution is shown in Fig. S5a (the same data as in the 3.3 ps frame in Fig. 1D). A synthetic background image was constructed by smoothing the data image column-by-column using a code based on a penalized least squares method. Then the quantity $\frac{n}{n_0} - 1$ that is proportional to the spatial gradient of the path-integrated magnetic field was calculated using Figs. S5a and b. By taking 1D Fourier analysis of each column of Fig. S5c, the time-resolved k spectrum of the magnetic field was calculated and is shown in Fig. S5d. Note that the constant coefficient has been dropped for clarity and this does not affect the extraction of growth rate. In Fig. S5e we show a representative lineout of the calculated magnetic field spectrum taken at $\frac{k_y}{2\pi} \approx 0.02\ \mu m^{-1}$ where the signal appears to grow the fastest. The signal (blue curve) clearly shows that the signal grows exponentially and has increased by more than a factor of ten within the delay range of 1-3 ps. The orange solid line represents the best fit to the data. The slope of the line gives the growth rate of the signal. By repeating the same analysis for every k_y (each row of Fig. S5d), the k-resolved growth rate of the magnetic field was retrieved and is shown in Fig. S5f. The error bars represent the 95% confidence interval of the fit. The curve shown here in Fig. S5f is the same as the green curve in Fig. 3E.

Plasma density

The anisotropic plasma in the experiment was produced by ionizing a supersonic gas jet emanating from a nozzle with 5-mm diameter opening by the CO2 laser. The density profile of the gas jet was characterized using an ionization-plasma-grating-based method in a separate laboratory. The results are shown in Fig. S6.

In this offline measurement, the backing pressure (87 psig) and the nominal height of the laser with respect to the nozzle exit (H=2.5 mm) were kept the same as in the ATF experiment. The laser was put in the center of the gas jet. The density profile of the neutral hydrogen gas jet corresponding to this condition is shown by the blue curve in Fig. S6. In the experiment, there are some uncertainties in determining the laser position with respect to the nozzle exit. To account for this, we have shifted the laser in either the vertical (H=3.0 mm) or horizontal (H=2.5 mm, X=0.5 mm) direction to verify the change in the plasma density. The results are shown by the orange and green curves, respectively. It is seen that the overall density profile remains similar and only the peak density varies. The average plasma density in the plateau region (e.g., averaged within $|x| \leq 0.6$ mm) is $(1.8 \pm 0.2) \times 10^{18}$ cm^{-3}.

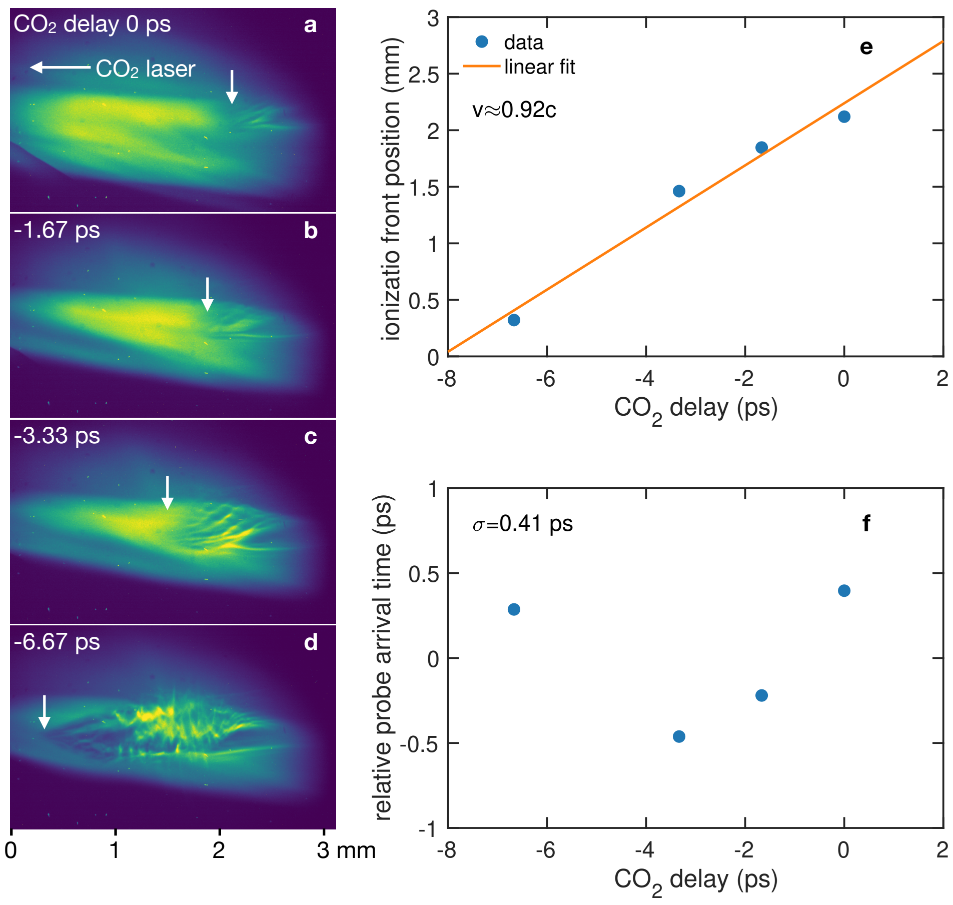

Fig. S1. Visualization of the propagation of the CO_2 laser ionization front and deduced timing jitter. (a)-(d) show four frames where the CO_2 laser delay is changed and the propagation of the ionization front from right to left is visible (in each frame the location of the ionization front is marked by the white arrow). (e) shows the ionization front position as a function of CO_2 delay. (f) The relative arrival time of the electron probe. Positive number means the probe arrives later.

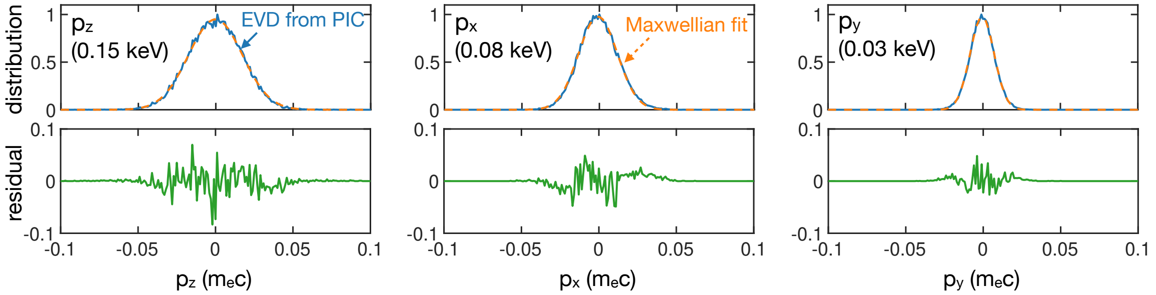

Fig. S2. EVDs from 3D PIC simulation and Maxwellian fits. The top row is reproduced from Fig. 1C. The bottom row shows the fit residuals for the three EVDs.

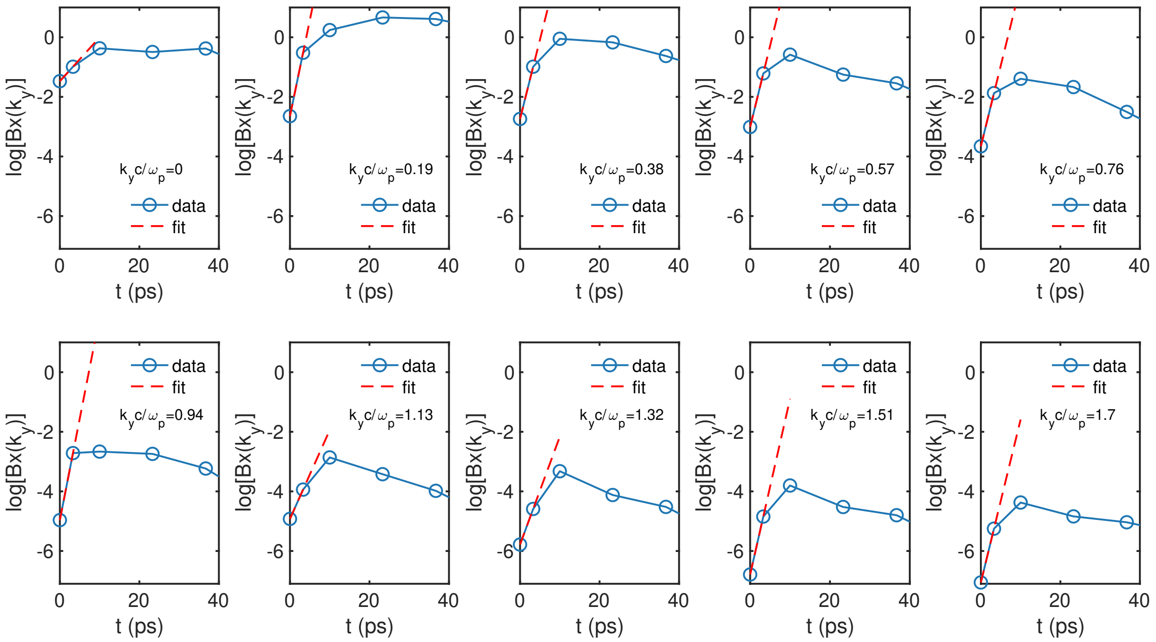

Fig. S3. Measured growth of the B_x field at several representative k_y (blue circles) and the exponential fit to the data (red dashed lines).

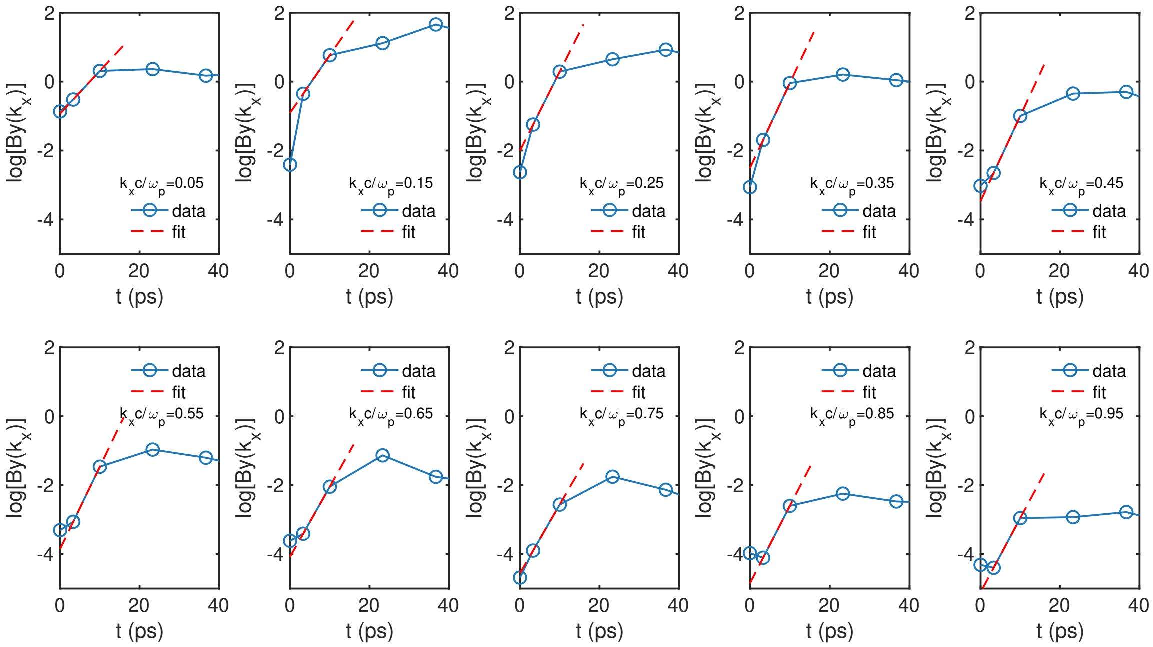

Fig. S4. Measured growth of the $\boldsymbol{B_y}$ field at representative $\boldsymbol{k_x}$ (blue circles) and exponential fit to the data (red dashed lines).

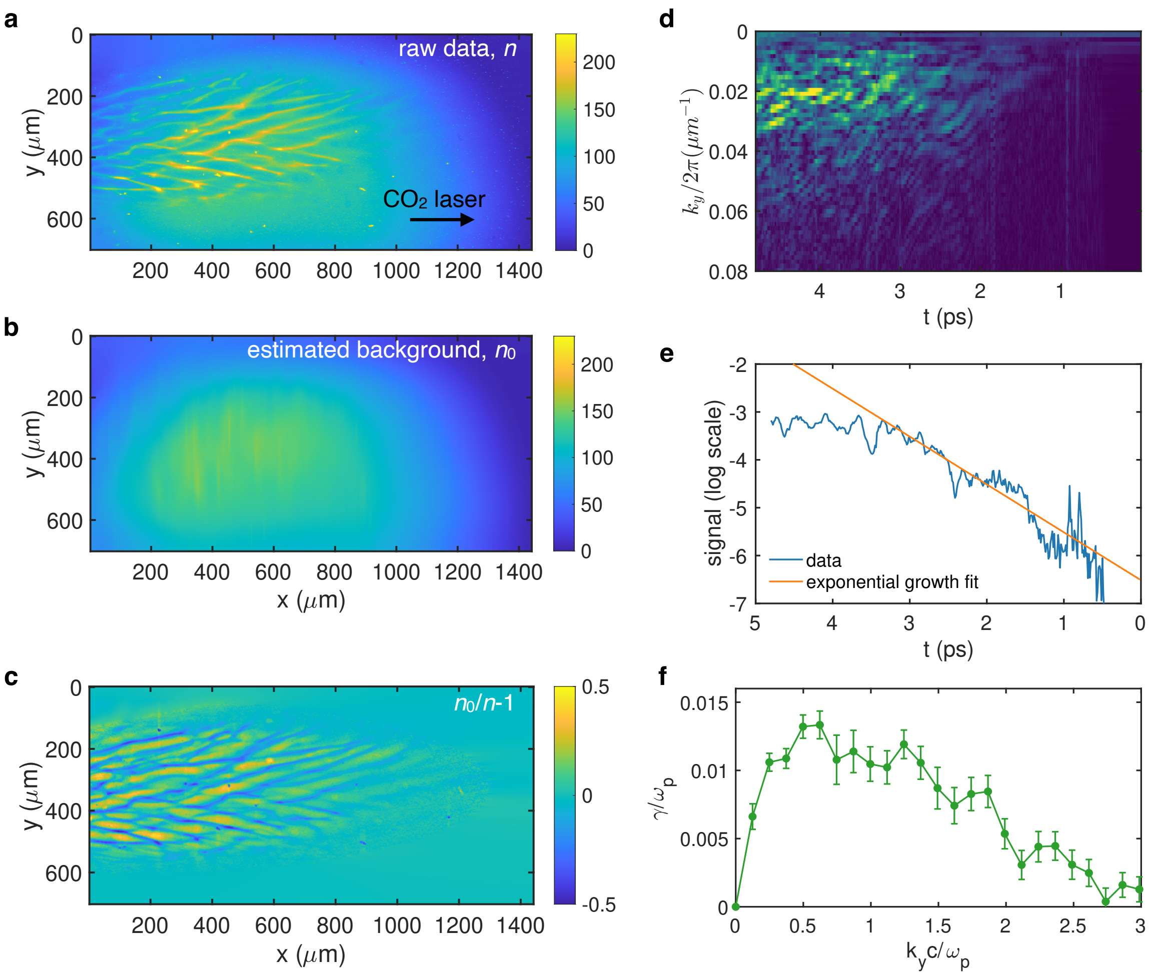

Fig. S5. Methodology used to deduce k-dependent growth rate of B_x using the measured modulated probe flux data. (a) Measured probe flux n (same as the 3.3 ps frame in Fig. 1D). (b) Estimated background n_0 by smoothing the data in (a) column-by-column. (c) The quantity $\frac{n_0}{n} - 1$ that is proportional to the gradient of the path-integrated magnetic field. (d) Calculated k_y spectrum of the B_x [i.e., $k_y \bar{B}_x(k_y)$] by taking 1D Fourier transform of (c) column-by-column. The horizontal axis has been converted to relative delay by setting the right edge of the image in c (x=1440) to be time zero. (e) A lineout of the spectrum taken at $\frac{k_y}{2\pi} \approx 0.02\ \mu m^{-1}$ (blue curve, in log scale). The orange line shows the exponential fit to the data within the range of 1-3 ps. (f) Retrieved k-resolved growth rate by performing similar analysis for all different k_y.This is the green curve in Fig 3E of the manuscript.

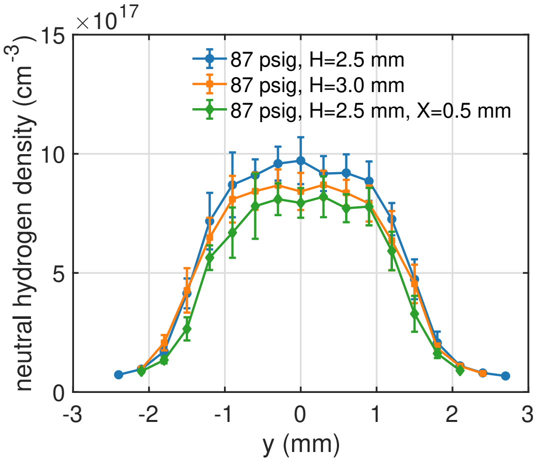

Fig. S6. Measured plasma density profile. The backing pressure is the same as in the experiment. The laser was put at the center of the gas jet and ~2.5 mm away from the nozzle exit, which corresponds to the H=2.5 mm line. The two other lines (H=3.0 mm and H=2.5 mm, X=0.5 mm) represent a 0.5 mm shift in either the vertical or horizontal direction to account for the uncertainty in determining the laser position with respect to the nozzle exit.

Movies of the electron probe density, retrieved magnetic fields and plasma current density

By changing the delay of the electron probe beam with respect to the CO_2 laser, a movie of the density bunching of the electron probe due to deflections caused by the Weibel magnetic fields was recorded, from which we retrieved the magnetic fields and plasma current density evolution. These movies are uploaded separately.

Movie S1 (separate file). Evolution of the measured bunching of electron probe.

Movie S2 (separate file). Evolution of the retrieved magnetic field components.

Movie S3 (separate file). Evolution of the retrieved plasma current density.
