# Supplementary figures and images for: Mapping the self-generated magnetic fields due to thermal Weibel instability
Source: Proc Natl Acad Sci U S A. 2022 Dec 5;119(50):e2211713119. doi: 10.1073/pnas.2211713119 (PMC9897480; doi:10.1073/pnas.2211713119)

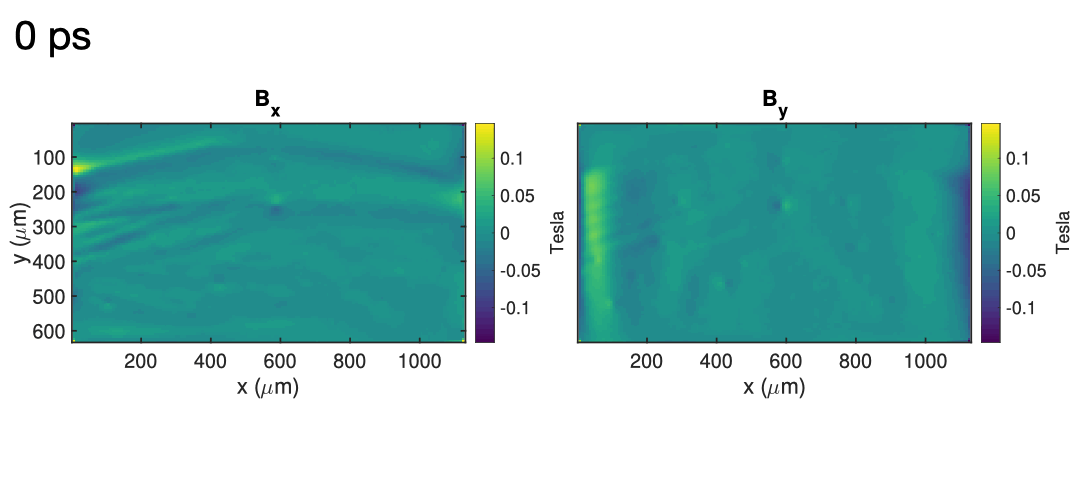

Supplement: Movie S1. — Evolution of the measured bunching of electron probe. [file pnas.2211713119.sm01.gif]

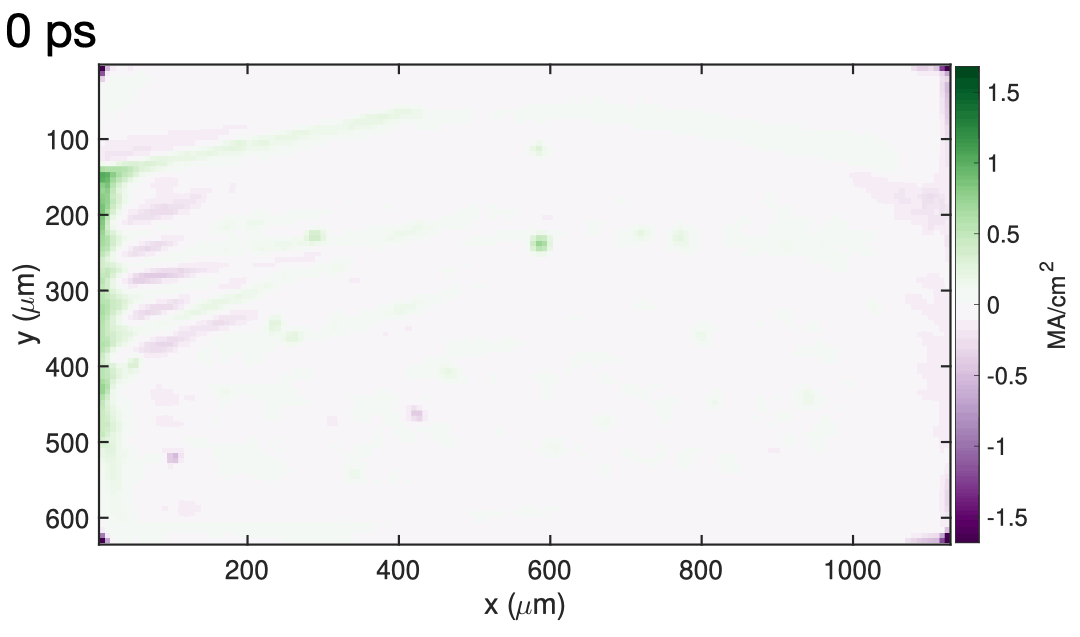

Supplement: Movie S2. — Evolution of the retrieved magnetic field components. [file pnas.2211713119.sm02.gif]

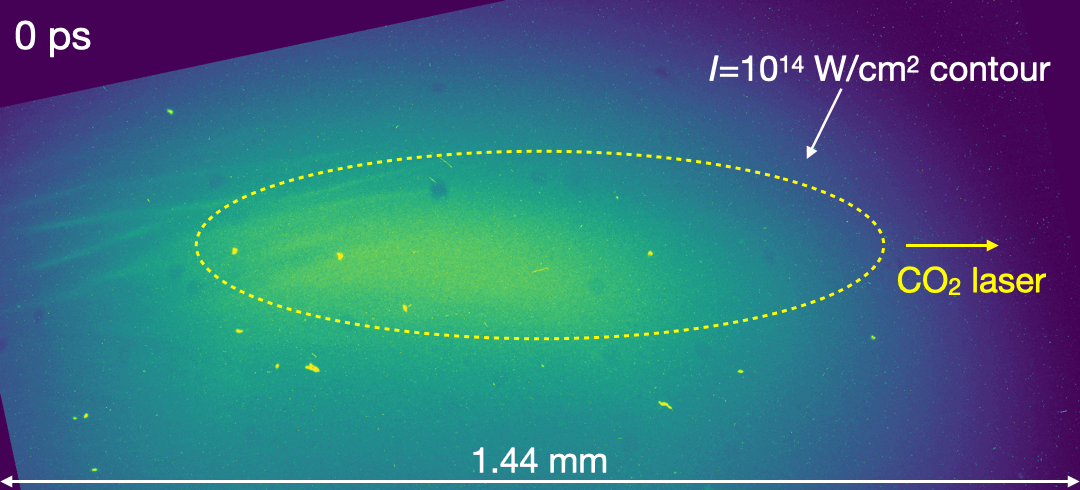

Supplement: Movie S3. — Evolution of the retrieved plasma current density. [file pnas.2211713119.sm03.gif]
